# Appearance of choline metabolites in plasma and milk when choline is infused into the abomasum with or without methionine

**DOI:** 10.3168/jdsc.2022-0359

**Published:** 2023-10-03

**Authors:** C.J.R. Jenkins, P.J. Kononoff

**Affiliations:** 1 Department of Animal Science, University of Nebraska–Lincoln, Lincoln, NE 68583; 2 Standard Dairy Consultants, Omaha, NE 68144

## Abstract

Four lactating, ruminally cannulated Jersey cows, (mean ± standard deviation) 264 ± 54.2 d in milk and 484 ± 24.1 kg of body weight, were arranged in a 4 × 4 Latin square design to measure the effects of abomasal infusion of choline chloride with or without dl-Met on milk and plasma choline metabolites and plasma AA in cows fed a Met-deficient diet. Cows were randomly assigned to 1 of 4 experimental treatments: (1) control; no supplemental Met or choline (CON), (2) 13 g/d of choline ion delivered via abomasal infusion (CHO), (3) 13 g/d of Met delivered via abomasal infusion (MET), and (4) 13 g/d of choline and 13 g/d of Met delivered via abomasal infusion (CHO + MET). Cows received the same basal diet throughout the experiment, which was formulated to be deficient in Met (−5.0 g of Met using the NASEM, 2021, model). Periods were 7 d in length with d 1 to 2 serving as a wash-out period and cows being infused on d 3 to 7. Milk samples were collected twice daily on d 5 to 7 and were analyzed for fat, true protein, lactose, and choline metabolites including betaine, phosphocholine, and free choline using hydrophilic interaction liquid chromatography-tandem mass spectrometry. Blood samples were collected via venipuncture of the coccygeal vein at 1100, 1300, and 1500 h on d 7 of each period and were analyzed for free AA as well as choline metabolites. Plasma Met increased in response to Met infusion and an interaction with choline and Met infusion was observed in the plasma concentration of branched-chain AA. Cows receiving choline exhibited the greatest Cho yield in milk. Milk phosphocholine yield tended to be highest when both choline and Met were infused.

## Highlights

• Milk choline yield increases when choline is infused into the abomasum.
• When dietary Met is deficient, choline may be used to regenerate Met.
• Phosphocholine as a marker for bioavailability of choline may be influenced by Met status.
• Plasma branched-chain AA concentration may be affected by choline and Met infusion.

## Graphical Abstract

**Figure:**
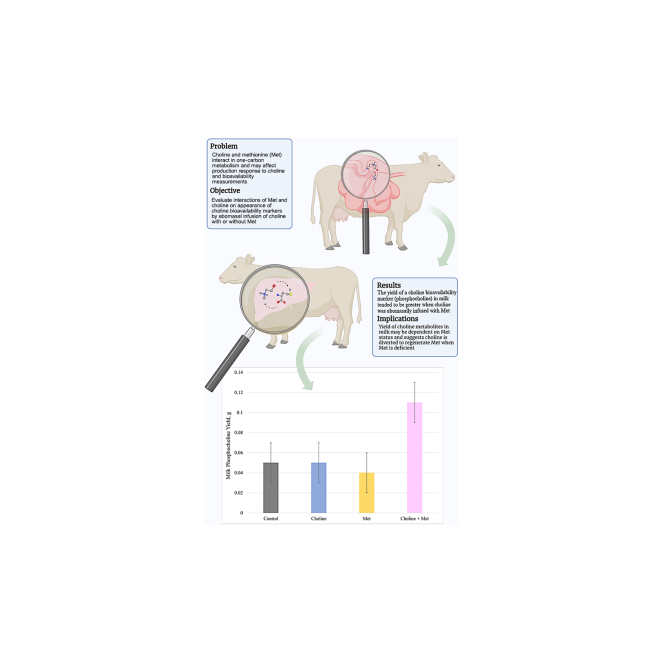
**Summary:** Choline chloride with or without methionine (Met) was infused into the abomasum of lactating dairy cows receiving a Met-deficient diet to investigate the effects on milk and plasma choline metabolites and amino acids (AA). Milk choline metabolites increased in response to choline infusion, and milk phosphocholine yield tended to be highest when both choline and Met were infused. The results demonstrated the potential impact of dietary Met level on choline bioavailability markers due to the interplay of choline and Met in one-carbon metabolism.

Choline and Met are both methyl donors involved in one-carbon metabolism, a variety of pathways that work together to promote lipid, nucleotide, and protein synthesis, as well as an abundance of methylation reactions and regulators of the animals' oxidative status (McFadden et al., 2020; White, 2020). Accordingly, supplemental choline and Met, provided as rumen-protected choline (**RPC**) and Met (**RPM**), have been explored as production- and health-enhancing feed additives in dairy cattle nutrition, particularly in periparturient cattle in the case of RPC. Furthermore, because they both participate in one-carbon metabolism, their interplay has been examined when fed in combination. For example, in a meta-analysis summarizing the effects of RPC supplementation (Arshad et al., 2020), positive milk production responses resulting from RPC were lessened as metabolizable Met was increased in the diet, suggesting that RPC is more beneficial when Met is limited in periparturient dairy cows. The researchers determined that when diets were low in metabolizable Met as a percent of MP (**METMP**; e.g., 1.80%), supplementing choline ion at a rate of 12.9 g/d increased milk yield by an average of 2.0 kg/d; however, when METMP was high (e.g., 2.30%), the same amount of choline supplementation resulted in an increase of 1.02 kg/d of milk. A similar pattern was observed for ECM yield (2.13 kg/d increment of ECM vs. 1.20 kg/d ECM in low- and high-METMP diets, respectively). There was also an interaction between the linear effects of choline and METMP on milk protein yield, where supplementing 12.9 g/d of choline ion when METMP was low resulted in an average increase in milk protein yield of 0.07 kg/d, but when METMP was high, increased 0.04 kg/d. In an experiment conducted by Potts et al. (2020), these interactions were explored directly in periparturient dairy cattle. Cows were fed RPC (13.0 g/d choline ion), RPM (9 g/d dl-Met prepartum and 13.5 g/d dl-Met postpartum), RPC and RPM (13.0 g/d choline ion and 9 g/d dl-Met prepartum and 13.5 g/d dl-Met postpartum), or control (no RPM or RPC) during the periparturient period. The Met concentrations were 1.77 and 1.82 Met, % of MP in pre- and postpartum diets, respectively. The researchers observed an 8.7 kg/d increase in milk yield for multiparous cows when RPC was fed without RPM, though the increase was not observed when RPC and RPM were fed in combination. Additionally, despite the notion that choline may have a sparing effect on Met and promote milk protein synthesis, RPC fed with RPM did not show any significant impact on milk protein concentration.

While the interplay of supplemental choline and Met on production performance has been explored, the effects of supplementation in combination on the appearance of choline metabolites and plasma Met levels has not been fully established. The objective of this experiment was to investigate potential changes in the appearance of choline metabolites in milk and plasma when choline was abomasally infused with or without infusion of Met. Choline metabolites appearing in plasma and milk have been utilized previously as bioavailability markers for choline (de Veth et al., 2016); therefore, the potential effects of Met supply on the appearance of choline metabolites should be considered. We hypothesized that in lactating cows fed a Met-deficient diet, the appearance of choline metabolites in milk and plasma resulting from choline infusion would be lower than when infused along with Met. Should this be observed, we speculate that at least some portion of choline is diverted for use a methyl donor to spare Met for milk protein synthesis.

Before conducting the experiment, procedures using animals were approved by the University of Nebraska–Lincoln Institutional Animal Care and Use Committee. Four lactating, ruminally cannulated Jersey cows, averaging (±SD) 264 ± 54.2 DIM at the start of the experiment and averaging 484 ± 24.1 kg of BW, were used in this experiment. Cows were housed in a temperature-controlled barn at the Dairy Metabolism Facility in the Animal Science Complex of University of Nebraska–Lincoln (Lincoln, NE) in individual tiestalls equipped with rubber mats, and were milked at 0700 and 1800 h. Cows were fed the same basal diet for 30 d before and throughout the experiment, which was formulated to meet expected dietary requirements determined using the NASEM (2021) model with the exception of Met. The ingredient and chemical composition of the TMR is listed in Table 1. The TMR was mixed daily and animals were fed once daily at 0930 h to target 5% refusals.

**Table 1:** Ingredient, chemical composition,^1^ and AA balance of the TMR

Composition	Value
Ingredient, % of DM	
Corn silage	43.2
Alfalfa hay	23.0
Corn grain, ground	11.5
Nonenzymatically browned soybean meal^2^	7.8
Soybean hulls, ground	6.9
Soybean meal	3.0
Calcium carbonate	1.2
Molasses, cane	1.1
Sodium bicarbonate	1.1
Dicalcium phosphate	0.4
Salt	0.3
Magnesium oxide	0.2
Tallow	0.2
Trace mineral and vitamin premix^3^	0.1
Chemical analysis, % of DM (unless otherwise indicated)	
DM	70.8
CP	14.8
Soluble protein	4.67
ADICP^4^	1.23
NDICP^4^	2.70
ADF	21.8
NDF	33.7
Lignin	3.55
Starch	25.7
Sugar	4.95
Ash	9.5
Ca, %	1.19
P, %	0.45
Mg, %	0.32
K, %	1.87
S, %	0.14
Na, %	0.44
Cl, %	0.44
Choline metabolites,^5^ mmol/kg	
Betaine	0.94
Choline	0.70
Phosphocholine	0.007
AA balance (modeled)^6^	
Met, g	−5.0
Met, % required	89.6
Met, % MP	2.19
Lys, g	0.0
Lys, % required	100.0
Lys, % MP	7.75
Lys:Met	3.53:1

1 Values determined by Cumberland Valley Analytical Services (Hagerstown, MD).

2 Soypass, LignoTech (Overland Park, KS).

3 Formulated to supply approximately 168.75 kIU/d vitamin A, 43.75 kIU/d vitamin D, 1,025 IU/d vitamin E, 16.7 mg/d Co, 182 mg/d Cu, 18.9 mg/d I, 7.27 mg/d Fe, 1,091 mg/d Mn, 5.96 mg/d Se, and 1,309 mg/d Zn in total rations.

4 ADICP = acid detergent insoluble crude protein; NDICP = neutral detergent insoluble crude protein.

5 Values determined by the University of Tennessee (Knoxville, TN).

6 Using the NASEM model (NASEM, 2021).

The experimental design was a 4 × 4 Latin square with 4 periods of 7 d. Cows received 1 of 4 experimental treatments that were designed to deliver choline ion, Met, or both via abomasal infusion. The experimental treatments were as follows: (1) control; no infusion of supplemental Met or choline (**CON**), (2) 13 g/d of choline ion (Thermo Scientific, Waltham, MA) delivered via abomasal infusion (**CHO**), (3) 13 g/d of Met (Sumitomo Chemical Company, Chuo-ko, Tokyo, Japan) delivered via abomasal infusion (**MET**), and (4) 13 g/d of choline ion and 13 g/d of Met delivered via abomasal infusion (**CHO + MET**). The abomasal infusion treatments were continuously infused each of the last 5 d of each period at a rate of 4 L per d (approximately 167 mL/h) using a peristaltic pump (Cole-Parmer Masterflex L/S, Vernon Hills, IL). Days 1 to 2 served as a wash-out period; we anticipated that 2 d of wash-out and 5 d of treatments would be sufficient for absorption of choline and incorporation into milk as described by de Veth et al. (2016). Choline ion was delivered as choline 99% and the molar proportion of choline ion in choline chloride is 74.6%, so 17.6 g/d was infused in solution to deliver 13 g/d postruminally. Methionine was delivered as dl-Met 99%, so 13.1 g/d was infused in solution to deliver 13 g/d postruminally. The abomasal infusion line was constructed according to Gressley et al. (2006). The infusion line and flange were passed through the ruminal cannula and set in place by hand through the omaso-abomasal orifice. To ensure the infusion line remained securely in the abomasum throughout each period, placement was evaluated manually on d 4 and 6. Approximately 2.5 kg of individual feed ingredients, as well as samples of the TMR for each treatment, were collected immediately after feeding on d 6 and 7. Samples of refusals were taken before feeding on d 6 and 7. Feed samples were frozen at −20°C for analysis as described in Morris and Kononoff (2021).

Milk yields were recorded daily throughout each period. Milk samples were collected twice daily for 3 consecutive days on d 5 to 7, placed in 50-mL conical tubes, and were immediately refrigerated at 3°C. At the end of each experimental period, milk samples were pooled by animal according to milk yield and were immediately frozen at −80°C before analysis of choline metabolites. The milk choline metabolites free choline (**Cho**), betaine (**Bet**), and phosphocholine (**Pcho**) were measured using hydrophilic interaction liquid chromatography-tandem mass spectrometry at an external laboratory (University of Tennessee, Department of Chemistry, Knoxville, TN). Additional samples were collected at the time of milking in 50-mL tubes containing 2-bromo-2-nitropropane-1,3 diol and shipped to DHIA (Heart of America DHIA, Manhattan, KS) where they were analyzed for fat, true protein, and lactose (AOAC International, 2000) using a B2000 Infrared Analyzer (Bentley Instruments, Chaska, MN). The yield of milk components were calculated using daily milk yield and component concentration on the day of collection. During the last 3 d of each period, milk yield was averaged and utilized in the statistical analysis.

Blood samples were collected via the coccygeal vein into Vacutainer tubes containing EDTA (Becton Dickinson Inc., Franklin Lakes, NJ) at 1100, 1300, and 1500 h on d 7 of each period. The samples were immediately placed on ice and centrifuged within 45 min at 4°C at 1,500 × *g* for 15 min. The supernatant was collected and 9-mL aliquots were placed into 10-mL conical tubes and stored at −80°C for later analysis of choline metabolites. Plasma choline metabolites (Cho, Bet, and Pcho) were measured using hydrophilic interaction liquid chromatography-tandem mass spectrometry at an external laboratory (University of Tennessee, Department of Chemistry, Knoxville, TN). An aliquot of 3 mL of plasma was deproteinized with 15% sulfosalicylic acid (4 parts plasma to 1 part 15% sulfosalicylic acid). Samples were placed in an ice bath for 10 min before centrifuging at 1,500 × *g* at 4°C for 20 min. The supernatant was collected, and 0.75-mL aliquots were placed into Nunc CryoTube vials (Nalge Nunc International, Roskilde, Denmark) and stored at −80°C. Plasma samples were submitted to the University of Missouri–Columbia Agricultural Experiment Station Chemical Laboratory for free AA analysis (Deyl et al., 1986; Fekkes, 1996). Plasma AA concentrations (μ*M*) were adjusted for the use of 15% sulfosalicylic acid.

Net portal flux of choline was predicted using milk choline metabolites according to de Veth et al. (2016) where net portal flux of choline (g/d) = −2.26 + 0.11[Bet]_Milk_ + 0.032[Pcho]_Milk_. Data were analyzed as a 4 × 4 Latin square using the PROC MIXED procedure of SAS (version 9.2, SAS Institute Inc., Cary, NC). The model fixed factors included the period, choline, methionine, and interaction between choline and methionine. Cow was considered the random effect. Treatment means were generated using the LSMEANS statement. Significance was declared at *P* < 0.05 and trends were defined as 0.05 < *P* ≤ 0.10.

The primary purpose of this experiment was to explore the potential changes in the appearance of choline metabolites in milk and plasma when choline was abomasally infused with or without Met in cows consuming a Met-deficient diet. We wished to investigate the potential impact of a Met-deficient diet on plasma free AA and the appearance of choline metabolites used as biomarkers (de Veth et al., 2016). Although narrow in scope, we sought to employ a simple approach to examine possible metabolic fates of methyl groups supplied from 2 different sources. In manipulation of both diet and supply conditions, we also chose to use cows in late lactation because the concentrations of choline metabolites in milk and plasma increase as lactation progresses (Artegoitia et al., 2014) and should increase the likelihood of detecting differences.

Plasma AA concentrations are listed in Table 2. The concentration of AA in blood can provide a gross indication of AA metabolism (Morris, 2020). The infusion of Met increased (*P* < 0.01) plasma Met, but no interaction (*P* = 0.68) between infused choline and Met was observed. Plasma Met concentration averaged 15.8, 15.3, 27.9, and 25.1 ± 3.20 μ*M* for CON, CHO, MET, and CHO + MET treatments, respectively. The results indicate that Met was successfully delivered postruminally under the conditions of the abomasal infusion system. Additionally, plasma Cys concentration was increased by infusion of Met (*P* < 0.01) and no interaction (*P* = 0.36) between choline and Met was observed. These results were likely related to the increased concentration of plasma Met observed, as Cys may be synthesized metabolically from dietary Met. This process occurs endogenously by first demethylating Met to homocysteine (via intermediate forms of S-adenosylmethionine and S-adenosylhomocysteine) and subsequent combination with Ser to yield cystathionine, which is then cleaved by cystathionase to yield Cys and α-ketoglutarate (Vasdev et al., 1999). An interaction of choline and Met infusion was observed in the plasma concentration of 2 branched-chain amino acids (**BCAA**), namely, Val (*P* = 0.02) and Leu (*P* = 0.01). These were reduced when comparing the CHO to the CON treatment, but were not different from MET and CHO + MET treatments. To our knowledge, this effect has not been observed in dairy cows; however, Alshaikh et al. (2016) observed a decrease in plasma BCAA concentration in children receiving a choline-enriched, structured lipid and Sivanesan et al. (2018) observed a reduction in plasma BCAA in choline-supplemented mice. Alshaikh et al. (2016) suggested that supplemental choline may have increased BCAA catabolism or increased uptake by the muscle. Increased uptake of BCAA by muscle in response to supplemental choline could partially explain increases in BW gain observed in cows receiving RPC during the periparturient period (Arshad et al., 2020). Based on the interactions we observed, the addition of Met may have reversed this effect of CHO on BCAA concentration. In the case of Leu, the concentration in the CON treatment was 160 ± 10.2 μ*M*, decreased (*P* = 0.05) to 122 ± 10.2 μ*M* when choline was infused, and returned to 149 ± 10.2 μ*M* when Met was infused with choline (*P* = 0.01). The plasma concentration of Lys was increased in cows infused with MET (*P* = 0.01), but no interaction between choline and Met was observed (*P* = 0.17). An interaction of choline and Met infusion was observed on the plasma concentration of Trp, which was greatest in cows receiving CHO + MET (*P* = 0.01).

**Table 2:** Plasma AA concentrations^1^ when lactating dairy cows were abomasally infused with choline, dl-Met, or a combination of choline and dl-Met

Item, μ*M*	Treatment^2^				SEM	*P*-value^3^		
	CON	CHO	MET	CHO + MET		Choline	Met	Choline × Met
Arg	63.3	57.7	67.3	66.4	4.31	0.42	0.14	0.55
His	42.9	41.0	41.5	46.8	4.26	0.43	0.33	0.13
Ile	128	105	118	124	7.83	0.25	0.50	0.06
Leu	160	122	143	149	10.2	0.05	0.47	0.01
Lys	74.4	66.5	81.2	83.4	6.57	0.42	0.01	0.17
Met	15.8	15.3	27.9	25.1	3.20	0.57	<0.01	0.68
Phe	51.5	41.6	51.1	48.1	3.17	0.07	0.33	0.27
Thr	85.8	76.6	82.9	94.2	6.58	0.81	0.11	0.04
Trp	35.3	34.5	34.7	38.1	1.97	0.10	0.07	0.02
Val	249	196	218	234	18.6	0.17	0.79	0.02
∑EAA^4^	905	757	865	909	57.0	0.17	0.14	0.03
Ala	185	178	200	199	12.8	0.58	0.03	0.68
Asn	36.1	35.2	35.5	43.0	2.91	0.26	0.22	0.16
Asp	3.39	1.43	1.30	1.09	1.19	0.42	0.36	0.51
Cit	70.7	70.4	68.8	74.8	5.00	0.18	0.53	0.16
Cys	15.8	16.1	18.0	17.4	0.72	0.69	<0.01	0.36
Gln	165	168	162	176	10.9	0.23	0.75	0.42
Glu	38.3	37.2	40.1	36.9	3.29	0.36	0.73	0.63
Gly	255	260	228	279	17.4	0.16	0.81	0.23
Orn	43.7	40.1	45.1	45.5	3.85	0.55	0.22	0.46
Pro	62.6	60.7	59.3	65.5	4.55	0.55	0.83	0.28
Ser	71.2	67.4	64.4	77.9	5.69	0.35	0.71	0.12
Tau	45.5	36.8	44.5	39.7	4.55	0.08	0.78	0.57
Tyr	46.6	39.9	42.1	48.4	3.10	0.95	0.53	0.08
∑NEAA^5^	1,039	1,012	1,009	1,103	38.9	0.34	0.38	0.11
∑TAA^6^	1,944	1,769	1,873	2,012	81.1	0.74	0.16	0.03
Carnosine	10.8	10.0	10.1	11.3	1.24	0.73	0.69	0.19
1-MH^7^	9.46	8.81	9.04	9.04	0.80	0.35	0.79	0.35
3-MH^7^	2.48	2.65	2.90	2.42	0.28	0.52	0.69	0.21
Urea	3,267	3,367	3,643	3,766	366	0.61	0.11	0.96

1 Values determined by University of Missouri–Columbia Agricultural Experiment Station Chemical Laboratories (Columbia, MO).

2 Treatments involved (1) no supplemental Met or choline, control (CON); (2) 13 g/d of choline ion delivered via abomasal infusion (CHO); (3) 13 g/d of Met delivered via abomasal infusion (MET); and (4) 13 g/d of choline and 13 g/d of methionine delivered via abomasal infusion (CHO + MET).

3 Main effect of treatments on plasma AA concentrations.

4 Sum of EAA (Arg, His, Ile, Leu, Lys, Met, Phe, Thr, Trp, and Val).

5 Sum of NEAA (Ala, Asn, Asp, Cys, Cit, Gln, Glu, Gly, Orn, Pro, Ser, Tau, and Tyr were considered as NEAA).

6 Sum of total AA.

7 MH = methylhistidine.

Overall DMI, milk yield, milk fat, and milk true protein measures were not different (*P* > 0.05) between treatments (Table 3). Due to the short duration and limited number of animals in this experiment, we did not expect to elicit a significant response on DMI, milk yield, or milk components. Plasma Bet significantly increased (*P* = 0.05) and Cho tended (*P* = 0.10) to increase with choline infusion. We did not observe an effect (P 0.11) on Pcho, which was expected as choline infusion typically results in increases in all 3 metabolites as observed by de Veth et al. (2016). Large variation and limited sample size in our study likely contributed to our inability to detect statistical differences in Pcho. We chose to infuse 13 g/d of choline ion in this experiment, which is similar to manufacturer recommendations for RPC products and similar to the level supplemented by Potts et al. (2020). Table 3 also lists the milk concentration (μ*M*) and yield (g/d) of Bet, Cho, and Pcho, and estimated net portal flux (g/d) of Cho. The concentrations of Bet in milk tended (*P* = 0.08) to increase with choline infusion, whereas Cho and Pcho were not affected (*P* ≥ 0.13) by treatments. Estimated net portal flux of choline tended (*P* = 0.08) to increase with choline infusion. The yield of milk Cho also increased (*P* = 0.04) in response to choline infusion, but not with Met infusion (*P* = 0.57); treatments were observed to be 0.09, 0.11, 0.08, and 0.15 ± 0.027 g/d for CON, CHO, MET, and CHO + MET, respectively. Several crossover designs have employed abomasal infusions to test the effects of choline or AA on plasma appearance (Rulquin and Kowalczyk, 2003; de Veth et al., 2016; Whitehouse et al., 2017; Coleman et al., 2019) and the number of animals used in these studies ranged from 3 to 10 cows. Given our limited resources and knowing that others had tested similar hypotheses with similar replication, we decided to use a 4 × 4 Latin square, yet our test may have lacked statistical power. Although not reported in the tables, observed error variance was 0.001174 and variance among experimental units or cows was 0.001671. In the future, this information, along with anticipated effect size, may prove of value to investigators conducting similar studies and could be used to calculate statistical power a priori using the procedures such as those outlined by Kononoff and Hanford (2006). Based on our statistical model, we did not observe an interaction (*P* = 0.12) between choline and Met infusion on milk Pcho yield. Using a post hoc LSMEANS test (superscripts not shown in tables), we tended to observe a difference (*P* = 0.06) between the CHO + MET versus CHO treatments (0.11 vs. 0.05 ± 0.02 g/d). This result is noteworthy in terms of our hypothesis in that when cows were fed a Met-deficient diet and infused with choline, the yield of Pcho in milk tended to be greater when choline was infused in combination with Met compared with choline alone. The response could provide preliminary evidence to the notion that choline may be diverted for use as a methyl donor to regenerate Met (Chandler and White, 2017; McFadden et al., 2020). This process may occur by the oxidation of choline to Bet, which subsequently combines with homocysteine to resynthesize Met. This reaction is catalyzed by the enzyme betaine-homocysteine *S*-methyltransferase (BHMT) and Coleman et al. (2019) observed increased BHMT activity in the liver of energy-deficient, lactating Holstein dairy cows receiving abomasal infusion of choline. Previously, de Veth et al. (2016) identified choline metabolites in milk that correlate strongly with net portal flux of choline, and practical results of our study show why investigators should be aware of the potential impact of dietary Met level on milk choline biomarker yield when comparing or designing bioavailability experiments that utilize these biomarkers to predict choline net portal flux.

**Table 3:** Intake, production, and milk concentrations and yields of betaine, choline, and phosphocholine, and predicted net portal flux of choline when lactating dairy cows were abomasally infused with choline, Met, or a combination of choline and Met and prediction of net portal flux of choline

Item	Treatment^1^				SEM	*P*-value^2^		
	CON	CHO	MET	CHO + MET		Choline	Met	Choline × Met
DMI, kg/d	20.1	22.2	22.2	23.5	1.12	0.12	0.11	0.66
Milk yield, kg/d	16.8	17.4	17.1	19.2	1.94	0.23	0.33	0.50
Milk fat, %	5.49	6.01	5.54	5.34	0.36	0.59	0.31	0.24
Milk fat yield, kg/d	0.91	1.05	0.95	1.02	0.101	0.11	1.00	0.52
Milk protein, %	3.88	3.91	3.94	3.89	0.122	0.80	0.87	0.41
Milk protein yield, kg/d	0.65	0.68	0.67	0.74	0.063	0.17	0.24	0.62
Milk lactose, %	4.53	4.52	4.50	4.51	0.071	0.83	0.49	0.67
Milk lactose yield, kg/d	0.76	0.79	0.77	0.87	0.099	0.26	0.38	0.49
Plasma,^3^ μ*M*								
Bet	63.0	151	49.6	105	29.8	0.05	0.35	0.62
Cho	0.52	0.75	0.47	0.61	0.096	0.10	0.34	0.65
Pcho	2.47	2.89	2.32	2.68	0.264	0.11	0.41	0.88
Milk,^3^ μ*M*								
Bet	78.6	155	68.9	137	35.7	0.08	0.71	0.91
Cho	55.5	58.9	54.6	71.6	12.96	0.13	0.35	0.28
Pcho	16.6	15.5	16.3	31.8	6.302	0.21	0.17	0.16
Milk yield,^3^ g/d								
Bet	0.15	0.33	0.11	0.32	0.092	0.07	0.77	0.86
Cho	0.09	0.11	0.08	0.15	0.027	0.04	0.57	0.15
Pcho	0.05	0.05	0.04	0.11	0.020	0.13	0.19	0.12
Net portal flux,^3^, ^4^ g/d								
Cho	7.00	15.4	5.91	14.0	4.00	0.08	0.76	0.97

1 Treatments involved: (1) no supplemental Met or choline, control (CON); (2) 13 g/d of choline ion delivered via abomasal infusion (CHO); (3) 13 g/d of Met delivered via abomasal infusion (MET); and (4) 13 g/d of choline and 13 g/d of Met delivered via abomasal infusion (CHO + MET).

2 Main effects of treatments on choline metabolite concentration and yield in milk and net portal flux predicted from milk choline metabolite concentrations.

3 Betaine (Bet), free choline (Cho), phosphocholine (Pcho).

4 Calculated according to de Veth et al. (2016), where net portal flux of choline (g/d) = −2.26 + 0.11[Bet]_Milk_ + 0.032[Pcho]_Milk_.

## References

[bib1] Alshaikh B., Schall J.I., Maqbool A., Mascarenhas M., Bennett M.J., Stallings V.A. (2016). Choline supplementation alters some amino acid concentrations with no change in homocysteine in children with cystic fibrosis and pancreatic insufficiency. Nutr. Res..

[bib2] AOAC International (2000).

[bib3] Arshad U., Zenobi M.G., Staples C.R., Santos J.E.P. (2020). Meta-analysis of the effects of supplemental rumen-protected choline during the transition period on performance and health of parous dairy cows. J. Dairy Sci..

[bib4] Artegoitia V.M., Middleton J.L., Harte F.M., Campagna S.R., De Veth M.J. (2014). Choline and choline metabolite patterns and associations in blood and milk during lactation in dairy cows. PLoS One.

[bib5] Chandler T.L., White H.M. (2017). Choline and methionine differentially alter methyl carbon metabolism in bovine neonatal hepatocytes. PLoS One.

[bib6] Coleman D.N., Vailati-Riboni M., Elolimy A.A., Cardoso F.C., Rodriguez-Zas S.L., Miura M., Pan Y.X., Loor J.J. (2019). Hepatic betaine-homocysteine methyltransferase and methionine synthase activity and intermediates of the methionine cycle are altered by choline supply during negative energy balance in Holstein cows. J. Dairy Sci..

[bib7] Deyl Z., Hyanek J., Horakova M. (1986). Profiling of amino acids in body fluids and tissues by means of liquid chromatography. J. Chromatogr. A.

[bib8] de Veth M.J., Artegoitia V.M., Campagna S.R., Lapierre H., Harte F., Girard C.L. (2016). Choline absorption and evaluation of bioavailability markers when supplementing choline to lactating dairy cows. J. Dairy Sci..

[bib9] Fekkes D. (1996). State-of-the-art of high-performance liquid chromatographic analysis of amino acids in physiological samples. J. Chromatogr. B Biomed. Appl..

[bib10] Gressley T.F., Reynal S.M., Olmos Colmenero J.J., Broderick G.A., Armentano L.E. (2006). Technical note: Development of a tool to insert abomasal infusion lines into dairy cows. J. Dairy Sci..

[bib11] Kononoff P.J., Hanford K.J. (2006). Technical note: Estimating statistical power of mixed models used in dairy nutrition experiments. J. Dairy Sci..

[bib12] McFadden J.W., Girard C.L., Tao S., Zhou Z., Bernard J.K., Duplessis M., White H.M. (2020). Symposium review: One-carbon metabolism and methyl donor nutrition in the dairy cow. J. Dairy Sci..

[bib13] Morris D.L. (2020).

[bib14] Morris D.L., Kononoff P.J. (2021). Dietary fatty acid and starch content and supplemental lysine supply affect energy and nitrogen utilization in lactating Jersey cows. J. Dairy Sci..

[bib15] NASEM. (National Academies of Sciences, Engineering, and Medicine) (2021).

[bib16] Potts S.B., Scholte C.M., Moyes K.M., Erdman R.A. (2020). Production responses to rumen-protected choline and methionine supplemented during the periparturient period differ for primi- and multiparous cows. J. Dairy Sci..

[bib17] Rulquin H., Kowalczyk J. (2003). Development of a method for measuring lysine and methionine bioavailability in rumen-protected products for cattle. J. Anim. Feed Sci..

[bib18] Sivanesan S., Taylor A., Zhang J., Bakovic M. (2018). Betaine and choline improve lipid homeostasis in obesity by participation in mitochondrial oxidative demethylation. Front. Nutr..

[bib19] Vasdev S., Ford C.A., Parai S., Longerich L., Gadag V. (1999). Dietary vitamin B_6_ supplementation attenuates hypertension in spontaneously hypertensive rats. Mol. Cell. Biochem..

[bib20] White H.M. (2020). ADSA Foundation Scholar Award: Influencing hepatic metabolism: Can nutrient partitioning be modulated to optimize metabolic health in the transition dairy cow?. J. Dairy Sci..

[bib21] Whitehouse N.L., Schwab C.G., Brito A.F. (2017). The plasma free amino acid dose-response technique: A proposed methodology for determining lysine relative bioavailability of rumen-protected lysine supplements. J. Dairy Sci..

